# Entre história e política: a construção da velhice e seus desafios para a Saúde Coletiva

**DOI:** 10.1590/0102-311XPT108026

**Published:** 2026-09-18

**Authors:** Daniela Bueno Barbosa, Bárbara Alves Pereira

**Affiliations:** 1 Universidade Federal de São Paulo, São Paulo, Brasil.; 2 Universidade Federal do Rio de Janeiro, Rio de Janeiro, Brasil.

O envelhecimento populacional é, hoje, um dos fenômenos demográficos mais relevantes do século XXI, com impactos profundos sobre os sistemas de saúde, as políticas sociais e as formas de organização da vida cotidiana. No Brasil, a transição demográfica ocorre de maneira acelerada: a redução das taxas de fecundidade e mortalidade, associada ao aumento da expectativa de vida, tem produzido o crescimento da população idosa, modificando o perfil epidemiológico do país e ampliando a demanda por serviços de saúde, cuidados de longa duração e políticas públicas. A velhice se torna uma questão central no campo da Saúde Coletiva, que passa a lidar com desafios como o aumento das doenças crônicas, incapacidades funcionais, dependência e desigualdades sociais no envelhecer. Nesse contexto, compreender a velhice implica reconhecer não apenas os processos fisiológicos do envelhecimento, mas também as condições sociais, culturais, econômicas e históricas que moldam as experiências de ser e se tornar velho em uma sociedade desigual como a brasileira.

É nesse cenário que está inserida a obra *Uma História da Velhice no Brasil*, de Mary Del Priore ^1^. Ao oferecer uma reconstrução histórica abrangente sobre os sentidos atribuídos à velhice desde o período colonial até a contemporaneidade, o livro permite compreender como as representações, os estigmas, as formas de cuidado e as políticas voltadas à população idosa foram sendo construídas ao longo do tempo. A obra fornece instrumentos analíticos fundamentais para que profissionais, pesquisadores e gestores da área da saúde reflitam criticamente sobre as bases culturais e morais que sustentam as atuais políticas de atenção à pessoa idosa no Brasil.

Mary Del Priore oferece uma reconstrução histórica das formas pelas quais o envelhecer foi percebido, vivido e significado no país, do período colonial ao século XXI. Nessa perspectiva - afinada à compreensão do envelhecimento como fenômeno historicamente produzido, conforme Stephen Katz ^2^ -, a velhice deixa de ser entendida como etapa natural e imutável da vida, configurando-se como uma condição socialmente construída, marcada por valores morais, arranjos familiares, práticas médicas e políticas públicas que variam ao longo do tempo.

Com base em vasta documentação - cartas, sermões, tratados médicos, crônicas, legislação, iconografia e literatura - Del Priore revela que, durante a Colônia e o Império, o envelhecimento era atravessado por forte ambiguidade. Se, por um lado, os idosos podiam ser associados à experiência, sabedoria e autoridade, por outro, eram frequentemente vinculados à decadência física, à improdutividade e à proximidade da morte. A velhice era menos uma categoria social definida e mais uma condição percebida a partir da perda da força de trabalho, da autonomia e da capacidade reprodutiva.

A autora mostra que, com a urbanização, a consolidação do trabalho assalariado e a emergência de discursos médicos no final do século XIX e início do XX, a velhice passa a ser progressivamente medicalizada e institucionalizada. O corpo velho se torna objeto de intervenções higienistas, e o envelhecimento começa a ser enquadrado como problema social, especialmente diante das transformações familiares e da redução da capacidade das redes domésticas de cuidado, no bojo da constituição de saberes gerontológicos voltados à regulação dos modos de envelhecer.

No decorrer do século XX, a criação da previdência social e das primeiras políticas públicas voltadas aos idosos redefine a velhice como categoria jurídica e administrativa. Del Priore evidencia que esse processo, embora tenha ampliado direitos e garantias, também reforçou a associação entre velhice, dependência e custo social, contribuindo para a consolidação de estigmas que ainda atravessam o imaginário contemporâneo.

Nos capítulos finais, a autora discute a emergência do discurso do “*envelhecimento ativo*”, da valorização da autonomia e da juventude prolongada, destacando como esses ideais convivem com profundas desigualdades sociais. Como observa Guita Grin Debert ^3^, a promessa de uma velhice saudável e independente tende a se restringir aos grupos com acesso continuado à educação, à renda e aos serviços de saúde, evidenciando a persistência de iniquidades estruturais no envelhecer brasileiro e a crescente responsabilização individual pelos modos de envelhecer.

A contribuição do livro para o campo da Saúde Coletiva é particularmente expressiva ao dialogar com a produção consolidada de Mary Del Priore sobre a história das sensibilidades, da família, do corpo e das idades da vida no Brasil. Em obras como *Histórias Íntimas: Sexualidade e Erotismo na História do Brasil* ^4^, *História das Mulheres no Brasil* ^5^ e *História do Amor no Brasil* ^6^, a autora já vinha demonstrando como as experiências corporais e afetivas são atravessadas por valores morais, normas sociais e dispositivos de poder. Em *Uma História da Velhice no Brasil* ^1^, Del Priore amplia esse campo de investigação ao inserir o envelhecimento como categoria histórica central, contribuindo de modo decisivo para os estudos que vêm problematizando a velhice no país, tradicionalmente dominados por abordagens biomédicas e demográficas. Ao evidenciar que as políticas de atenção à pessoa idosa não podem ser compreendidas apenas como respostas técnicas ao envelhecimento populacional, a autora insere sua análise em um diálogo consistente com a produção brasileira que vem problematizando a velhice como construção social, em especial com os trabalhos de Guita Grin Debert ^3^ e Júlio Assis Simões ^7^, entre outros autores que evidenciam como o envelhecer é atravessado por valores morais, hierarquias sociais e regimes de normalização dos corpos.

Tal perspectiva está alinhada aos aportes da Saúde Coletiva ao conceber o envelhecimento como fenômeno social complexo, estruturado por desigualdades de classe, gênero e raça, e ao problematizar criticamente os modelos normativos que orientam a organização dos serviços e as práticas de cuidado, exigindo abordagens que ultrapassem o paradigma biomédico e ampliem o debate sobre direitos, cidadania e modos de viver a velhice no Brasil.

No campo da Saúde Coletiva, *Uma História da Velhice no Brasil* desempenha um papel estratégico ao ampliar o repertório interpretativo do envelhecimento para além dos indicadores epidemiológicos e das respostas programáticas. Essa inflexão não é apenas analítica: orienta a organização dos serviços e as práticas de cuidado, frequentemente ancoradas em modelos normativos de funcionalidade e independência. Nesse quadro, a velhice é simultaneamente reconhecida como sujeito de direitos e constituída como categoria de intervenção, atravessada por discursos que classificam, hierarquizam e regulam os modos de envelhecer. Evidencia-se, assim, que as políticas públicas voltadas à população idosa não se limitam à proteção social, mas também participam da produção e legitimação de determinados modos de viver a velhice no Brasil.

Seria interessante, ainda, que a análise dialogasse de forma mais aprofundada com experiências de envelhecimento marcadas por desigualdades de raça, classe e território, especialmente em contextos periféricos e rurais, nos quais os sentidos da velhice e as formas de cuidado assumem configurações específicas. Tal ampliação poderia potencializar o alcance interpretativo da obra ao aproximar sua reconstrução histórica dos desafios contemporâneos do Sistema Único de Saúde (SUS) e das políticas públicas, voltadas à população idosa. Nesse sentido, o livro abre um campo fecundo para investigações que articulem história, desigualdades estruturais e práticas de cuidado, contribuindo para o aprofundamento do debate sobre o envelhecimento no Brasil em suas múltiplas dimensões.

Além dos aspectos já sublinhados, a obra contribui para qualificar a formulação, a avaliação e a implementação de políticas públicas ao revelar as raízes históricas dos modelos de cuidado, das representações sociais do idoso e das práticas institucionais que ainda orientam a organização dos serviços de saúde. Ao iluminar os sentidos culturais que estruturam as formas de cuidar, a autora fornece subsídios fundamentais para a construção de ações mais sensíveis às trajetórias de vida, às desigualdades sociais e às experiências concretas da população idosa. Ler este livro é, em última instância, reconhecer que não há política de saúde eficaz para a velhice sem uma compreensão profunda da história que moldou quem são - e como são tratados - os velhos no Brasil.
